# Transcriptome and Gene Editing Analyses Reveal *MOF1a* Defect Alters the Expression of Genes Associated with Tapetum Development and Chromosome Behavior at Meiosis Stage Resulting in Low Pollen Fertility of Tetraploid Rice

**DOI:** 10.3390/ijms21207489

**Published:** 2020-10-11

**Authors:** Zijun Lu, Xiaotong Guo, Zhiyu Huang, Juan Xia, Xiang Li, Jinwen Wu, Hang Yu, Muhammad Qasim Shahid, Xiangdong Liu

**Affiliations:** 1 State Key Laboratory for Conservation and Utilization of Subtropical Agro-Bioresources, South China Agricultural University, Guangzhou 510642, China; zjlu@stu.scau.edu.cn (Z.L.); xtguo@stu.scau.edu.cn (X.G.); zyhuang@stu.scau.edu.cn (Z.H.); xj516025647@163.com (J.X.); xiangli@scau.edu.cn (X.L.); jwwu@scau.edu.cn (J.W.); hyu@stu.scau.edu.cn (H.Y.); 2 Guangdong Provincial Key Laboratory of Plant Molecular Breeding, South China Agricultural University, Guangzhou 510642, China; 3 College of Agriculture, South China Agricultural University, Guangzhou 510642, China; 4 Guangdong Laboratory for Lingnan Modern Agriculture, South China Agricultural University, Guangzhou 510642, China

**Keywords:** autotetraploid, middle layer, tapetum, meiosis, pollen sterility

## Abstract

Autotetraploid rice is a useful rice germplasm for polyploid rice breeding. However, low fertility limits its commercial production. A neo-tetraploid rice with high fertility was developed from the progenies of crossing between autotetraploid lines by our research group. Our previous study showed that a myeloblastosis (MYB) transcription factor, *MOF1*, might be associated with the pollen development in tetraploid rice. However, little information is available about its role in pollen development in tetraploid rice. Here, we identified a new haplotype of *MOF1* from neo-tetraploid rice and marked it as *MOF1a*. Transcriptome and qRT-PCR analysis demonstrated that *MOF1a* highly expressed in anthers, and displayed differential expression in neo-tetraploid rice compared to tetraploid rice line with low pollen fertility. The mutant (*mof1a*) of *MOF1a*, which was generated by CRISPR/Cas9 knockout in neo-tetraploid rice, showed low pollen fertility, and also exhibited abnormal tapetum and middle layer development, and defective chromosome behaviors during meiosis. A total of 13 tapetal related genes were found to be up-regulated in meiotic anthers of *MOF1a* compared with wild type plants by RNA-seq analysis, including *CYP703A3*, *PTC1*, and *OsABCG26*, which had been demonstrated to affect tapetal development. Moreover, 335 meiosis-related genes displayed differential expression patterns at same stage, including nine important meiosis-related genes, such as metallothionein *OsMT1a*. These results demonstrated that *MOF1a* plays an important role in pollen development and provides a foundation for understanding the molecular mechanism underlying *MOF1a* in reproduction of tetraploid rice.

## 1. Introduction

Rice (*Oryza sativa* L.) is one of the most important food crops around the world. Rice cultivars are constantly improved to ensure food security, which largely depends on abundant germplasm resources. To enrich breeding germplasms of rice, autotetraploid rice has been developed through colchicine-mediated chromosome doubling, which showed great biological advantages, such as large kernels, stress resistance, and high heterosis [1,2,3,4]. However, it is hard to be used in commercial production because of its low seed setting [4,5,6].

Autotetraploid rice has complicated defects in reproduction, including abnormal pollen development, embryo sac development, double fertilization, embryogenesis, and endosperm development. High frequent pollen mother cells (PMCs) with abnormal chromosome behaviors and abnormal microtubule pattern, microspores with abnormal vacuolization or degeneration, and defective tapetum were observed in autotetraploid lines [4,7,8,9,10]. Abnormal development of embryo sac, embryogenesis, and endosperm also resulted in no fertilization and low seed setting in autotetraploid lines [11,12,13,14]. Polyploidization alters expression patterns of genes, miRNA expression, and methylation during the development of anther, embryo sac, and endosperm [4,10,15,16,17], and enhances pollen sterility loci interactions in intersubspecific hybrids [5,18,19,20].

In this case, it was meaningful to create high fertility tetraploid rice germplasm, which could be achieved through genetic improvement [21,22]. To solve the issue of low fertility in autotetraploid rice, our research team developed a new type of tetraploid rice named “neo-tetraploid rice” and reported six neo-tetraploid lines, including Huaduo1 to Huaduo5 and Huaduo8. Neo-tetraploid lines display normal morphological traits and high fertility and can overcome sterility in autotetraploid rice. Moreover, hybrids displayed strong heterosis, which were developed from neo-tetraploid lines crossed with autotetraploid lines with low fertility, including T452×Huaduo3, T449×Huaduo1, and T485×Huaduo8 [1,18,23,24,25]. Therefore, understanding important genes involved in reproduction of this new germplasm, neo-tetraploid rice, will contribute to promote polyploid rice breeding.

Anther or pollen development is important for reproduction and generation alternations of rice. Meiosis is crucial for pollen development, involving more than 5000 meiosis stage-related genes [26,27,28,29,30,31,32,33,34,35]. Our previous research identified a myeloblastosis (MYB) family transcription factor, *LOC_Os04g47890*, which might play an important role in the pollen development of autotetraploid rice [10]. Recently, *LOC_Os04g47890* was found to regulate spikelet development, including size and number of floral organs, and was named *MOF1* [36]. However, little information about *MOF1* affecting pollen development was available both in diploid and tetraploid rice.

In this study, we further found that *MOF1* differentially expressed in one neo-tetraploid line, Huaduo1 (H1), and a high fertility recombinant inbred line, HF, compared to low fertility line, LF, by using RNA-seq. Then, CRISPR/Cas9 was employed to edit *MOF1* in H1. As expected, the mutant lines exhibited abnormal pollen development and low seed sets. Cytological observations displayed that *MOF1* affects tapetum development and chromosome behavior. Transcriptomic analysis revealed numbers of differentially expressed genes associated with tapetum and meiosis in mutant plants. These results indicate that *MOF1* plays an important role in pollen development and provide a foundation for understanding the molecular mechanism of *MOF1* in male reproduction of neo-tetraploid rice.

## 2. Results

### 2.1. RNA-seq Analysis Revealed Differential Expression of MOF1 in Neo-Tetraploid Rice Compared to the Recombinant Inbred Line with Low Fertility

To understand the difference in gene expression patterns between tetraploid lines with different fertility levels, RNA-seq was employed to analyze global genes expression in the anthers during meiosis using neo-tetraploid rice, H1 with 95.15% pollen fertility, and two recombinant inbred lines, one with high pollen fertility (HF, 85.58%) and another with low pollen fertility (LF, 54.82%) (Table 1).

More than 475 million clean reads were detected from each sample. The average percentage of mapped reads to reference genome was 94.54% (Table S1). There was a high degree of correlation between three biological replicates of RNA-seq data in HF, LF, and H1 with a Pearson correlation coefficient more than 0.888 (Figure S1).

**Figure 1:**
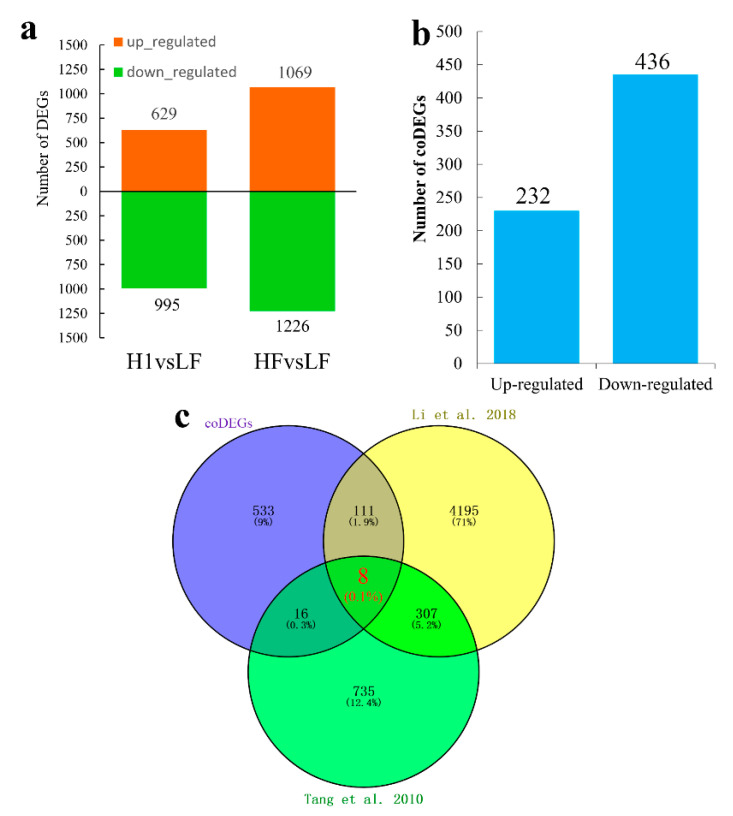
Identification of candidate genes associated with anther wall development by RNA-seq. (**a**) The number of differentially expressed genes (DEGs) during microsporogenesis. (**b**) The quantity of common differentially expressed genes (coDEGs) between HF and H1. (**c**) Venn analysis of coDEGs, DEGs in 02428-4x relative to 02428-2x [10] and DEGs preferentially expressed during meiosis [33]. Eight genes (red) overlapped among the three group DEGs.

A total of 1624 differentially expressed genes (DEGs) were identified between H1 and LF, including 629 up- and 995 down-regulated genes (Table S2). In addition, 2295 genes were designated as DEGs between HF and LF, including 1069 up- and 1226 down-regulated genes (Figure 1a, Table S3). Furthermore, there were 668 common DEGs (hereafter referred as coDEGs) shared by HF and H1, including 232 up- and 436 down-regulated coDEGs (Figure 1b, Table S4). Among these 668 coDEGs, there were eight special genes that were identified as DEGs in anther of 02428-4x (low fertility) relative to 02428-2x (normal fertility) [10] and specifically or preferentially expressed in PMCs [33], including *LOC_Os01g27340*, *LOC_Os01g50310*, *LOC_Os03g59880*, *LOC_Os03g61670*, *LOC_Os04g47890*, *LOC_Os08g37456*, *LOC_Os08g43240*, and *LOC_Os10g05180* (Figure 1c, Table S5). We compared the expression levels of these eight genes with previously available microarray and RNA-seq data associated with anther meiotic stage-related expression in different ploidy rice [10,26,33,34,35,36], and inferred that *LOC_Os04g47890*, which was named *MOF1* [36], may play an important role in pollen development in neo-tetraploid rice.

### 2.2. High Expression of MOF1 Was Detected in Anther at the Meiosis Stage

Annotated by Rice Genome Annotation Project Database [37], *MOF1* is comprised of 3887 base pair (bp) that included six exons and five introns. The coding sequence of *MOF1* is 921 bp, and it encodes proteins with 306 amino acids, including an MYB DNA-banding domain. Resequencing and sanger sequencing showed a total of 20 single nucleotide polymorphisms (SNPs) or loci with insertion or deletion (InDels) were detected in *MOF1* of H1 compared to reference genome (Nipponbare, *Oryza sativa* L. ssp. *japonica*), including two important SNPs (1078th thymine and 3463th guanine) in CDS, four loci in the 5′ UTR region, and 14 loci in introns (Table S6). Two SNPs in CDS was further verified by cloning *MOF1* coding sequence from RNA of H1 anthers. The splicing of *MOF1* mRNA was consistent with the reference genome. This result indicates that *MOF1* in H1 encodes a protein with two different amino acids (80th Valine and 222th Serine) compared to Nipponbare (Glycine and Asparagine, respectively). This haplotype of *MOF1* in H1 was designated as *MOF1a* in this study.

**Figure 2:**
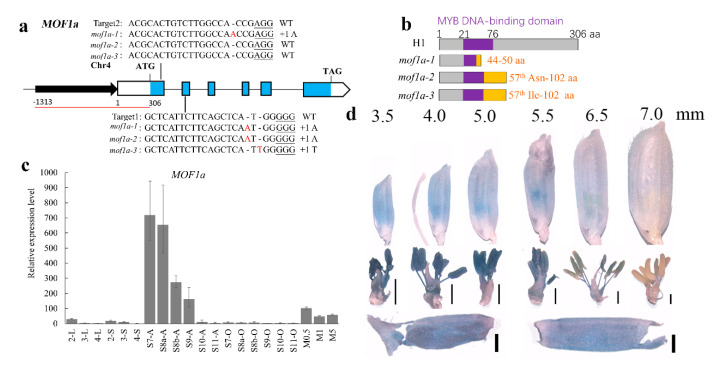
Gene information and expression pattern of *MOF1a* in H1. (**a**) A schematic diagram of the *MOF1a* gene bearing two CRISPR–Cas9 target sites indicated by black lines. UTRs, CDS regions, and introns are represented by white boxes, blue boxes, and lines, respectively. The two sides of the diagram show the alignment of wild-type (WT), *mof1a-1*, *mof1a-2,* and *mof1a-3* sequences containing the CRISPR–Cas9 target sites. The 20 bp CRISPR–Cas9 target sequences adjacent to the underlined protospacer adjacent motifs (PAMs) are indicated in the WT sequences. The newly created *mof1a-1*, *mof1a-2,* and *mof1a-3* mutants contain a 2 bp insertion of A, 1 bp insertion of A and 1 bp insertion of T (red), respectively. The region from −1313 to 306 bp covered by red line indicate the promoter of *MOF1a* from H1 for *MOF1a* promoter-β-glucuronidase (GUS) vector (pMOF1a::GUS). (**b**) Schematic diagrams of MOF1a proteins in H1 and *mof1a*. aa, amino acids. The orange panels (right) indicate predicted mutant protein fragment of *mof1a-1*, *mof1a-2,* and *mof1a-3* according to their new genotypes. The MOF1a proteins in *mof1a-1*, *mof1a-2,* and *mof1a-3* only keep 43 aa, 56 aa, and 56 aa as same as the MOF1a protein sequence of wild type plants. (**c**) Quantitative real-time PCR analysis of *MOF1a* expression in various tissues of H1 plant. Results are normalized against the expression levels of *ubiquitin* and shown as relative values to the lowest level set to 1. means (bars) and standard error (error bars) of three independent biological replicates are shown. 2, S8a; 3, S11; 4, 5 days after flowering; L, leaf; S, sheath; A, anthers; O, ovaries; S7 to S11, stages of anther development; M0.5, panicle with 0.50 cm length; M1, panicle with 1.00 cm length; M5, panicle with 5.00 cm length. (**d**) GUS staining of pMOF1a::GUS lines shows that *MOF1a* highly expresses in stamen, palea, and lemma. The numbers on the top side indicate the length of observed spikelets.

In order to make sure the spatial and temporal expression of *MOF1a*, three internet tools (eFP Browser [38], RiceXPro [39], and GEO Profiles of NCBI) and a total of 84 RNA-seq datasets were analyzed and *MOF1* displayed high expression in anthers at meiosis stage (Figure S2, Figure S3). Moreover, qRT-PCR demonstrated high transcriptional level of *MOF1a* during anther development in H1, which was consistent with the RNA-seq data and three internet tools. Meanwhile, qRT-PCR results also displayed that the expression level of *MOF1a* in anthers continuously decreased from S7 to S11 stage (Figure 2c).

Again, the *MOF1a* promoter-β-glucuronidase (GUS) vector (pMOF1a::GUS) was constructed and transformed into Nipponbare calli, in which the 1619 bp sequence ahead of initiation codon of *MOF1a* cloned from H1 plant was used as *MOF1a* promoter. In agreement with qRT-PCR results, strong GUS signals were detected in anthers and continuously decreased during anther development (spikelet length from 3.5 mm to 6.5 mm). During these stages, GUS signals were also detected in stamen, lemma, and palea, but not in lodicules, pistil, sterile lemma, and rudimentary glume. The GUS signal became almost undetectable in spikelets with 7.0 mm length (Figure 2d). Taken together, *MOF1a* was highly expressed in stamen and its expression level decreased during stamen development.

### 2.3. The Mutants with Low Pollen Fertility Were Obtained by CRISPR/Cas9 in Neo-Tetraploid Rice

To understand the reproductive role of *MOF1a*, this gene was edited in the neo-tetraploid rice, H1, using the CRISPR/Cas9 editing system. A total of 34 T_0_ transgenic plants were obtained, and three homozygous mutants (*mof1a*) with frameshift mutation in the targeted loci, including *mof1a-1*, *mof1a-2,* and *mof1a-3,* were successfully selected from T_1_. Both target loci were inserted 1 bp ‘A’ (adenine) in *mof1a-1*; 1 bp ‘A’ inserted into target1 of *mof1a-2*; 1 bp ‘T’ (thymine) inserted into target1 of *mof1a-3*; no polymorphism was detected in target2 of *mof1a-2* and *mof1a-3* (Figure 2a). While predicting the protein sequence of MOF1a in *mof1a* according to their new genotypes, the MOF1a proteins in *mof1a-1*, *mof1a-2,* and *mof1a-3* only keep 43 amino acids (aa), 56 aa, and 56 aa as same as the MOF1a protein sequence of wild type plants. The premature translation termination codons were detected at the 51st codon in *mof1a-1* and the 103rd codon in *mof1a-2* and *mof1a-3* (Figure 2b)**.** *mof1a-3* is T-DNA free. Five randomly selected T_1_ *mof1a* plants from five T_0_ lines and fifteen T_2_ plants from *mof1a-1*, *mof1a-2,* or *mof1a-3* were used for phenotyping (Table S8).

**Figure 3:**
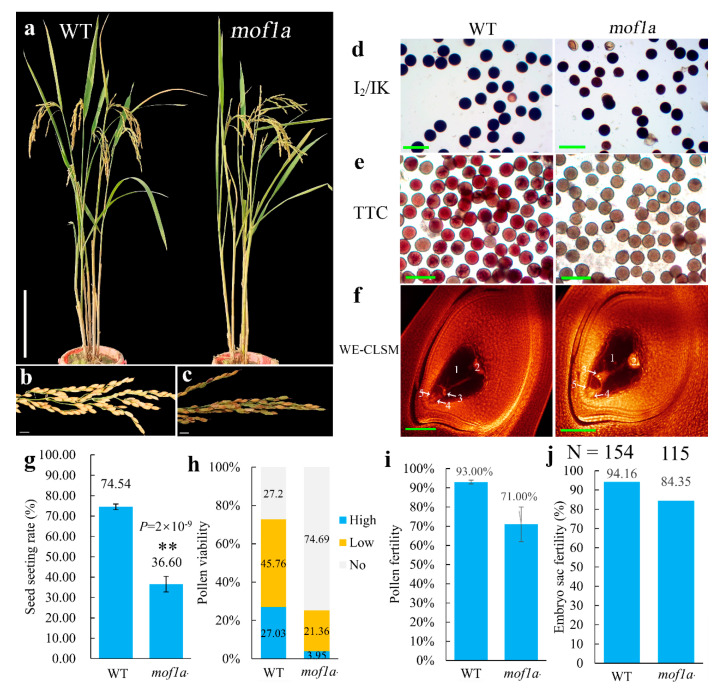
Morphological and cytological identification of *MOF1a*. (**a**) The plant type of H1 (WT) and *mof1a*. Bars = 20 cm. (**b**,**c**) Mature panicles of WT/*mof1a*. Bars = 1 cm. (**d**,**e**) I_2_/IK staining and TTC staining of WT/*mof1a* pollen grains at mature stage. (**f**) Mature embryo sac of WT/*mof1a*. 1, central cell; 2, antipodal cells; 3, polar nuclei; 4, synergid; 5, egg cell. Green bars = 100 µm. (**g**) Seed setting of WT and *mof1a*. Five plants of WT and *mof1a* were studied, respectively. *P* value was calculated using two-tailed Student’s *t*-test. “**” indicates that *P* value was less than 0.01. (**h**,**i**) Pollen viability (TTC) and pollen fertility (I_2_/IK) of WT and *mof1a* based on three independent biological samples. (**j**) The mature embryo sacs fertility of WT and *mof1a*. *n*, numbers of embryo sacs observed. The error bars indicate the ±SE.

The plant type of *mof1a* in T_1_ and T_2_ was similar to WT (Figure 3a). However, the seed setting of *mof1a* plants was only 36.60%, which was significantly lower than WT (74.54%). To reveal the reproductive defects of *mof1a*, we investigated the mature embryo sac, pollen fertility, and pollen viability of *mof1a* and WT. The mature embryo sac fertility of *mof1a* mutants was 84.35%, which was no different compared to WT (94.16%). The pollen fertilities of *mof1a* and WT plants were 71.00% and 93.00%, respectively. Further staining the intravital pollen grains of *mof1a* by 2, 3, 5-Triphenyl-2H-tetrazolium chloride (TTC) solution displayed that only 21.36% pollen grains could be stained with light reddish color and 3.95% showed a deep red color, while this was 45.76% and 27.03% for WT plants, respectively (Figure 3). These results indicate that low pollen fertility was the main reason for the low seed set of *mof1a,* and *MOF1a* might be involved in pollen development.

### 2.4. MOF1a Defect Affects Tapetum Development and Chromosome Behavior at Meiosis Stage of Tetraploid Rice

**Figure 4:**
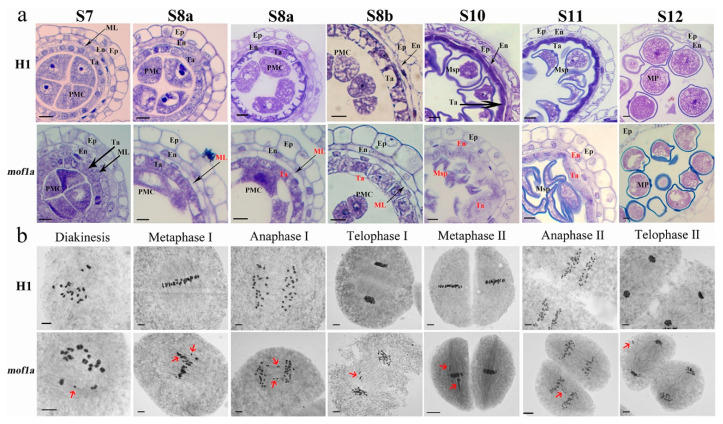
Anther transverse section analysis (**a**) and meiosis process (**b**) of WT and *mof1a.* Ep, epidermis; En, endothecium; ML, middle layer; Ta, tapetum. Red arrows indicate abnormal chromosome. Bars = 20 μm.

In anther transverse sections, no obvious differences were observed between WT and *mof1a-1* during S7 stage. The middle layer in WT displayed complete degeneration and became thick-lined structure at S8a stage. However, the anther walls of *mof1a-1* from S8 to S10 stage exhibited delayed the degeneration of the middle layer, which kept a cellular shape. Moreover, the tapetum cells of WT formed vacuoles and their stained areas reduced to form a cupped shape. At the S10 and S11 stage, tapetum cells degraded and differentiated. By contrast, the tapetum cells of *mof1a-1* did not form a special structure, as WT did, and kept a large shape without reduction of stained areas during the S8 stage. During the S10 to S11 stage, the tapetum cells were lightly stained and displayed inward hyperplasia in *mof1a-1*; at this stage, the round shape stained materials surrounding microspore were not found in *mof1a-1*, which was different from WT. At the S12 stage, pollen grains of *mof1a-1* still contained unfilled areas, while WT pollen grains were filled with lipid and starch (Table 2, Figure 4a).

During meiosis, H1 exhibited a lower number of abnormalities, which were similar to normal diploid rice. However, great changes were found in meiosis process of *mof1a-1*. At diakinesis, no difference was found in quantity or distribution of quadrivalents between H1 and *mof1a*, but the proportion of PMCs that were univalent or trivalent, was raised from 16.55% (145 observed WT PMCs) to 47.17% (104 observed *mof1a-1* PMCs). From metaphase I to telophase II, more PMCs with abnormal chromosome behavior were observed in *mof1a-1*, including chromosome dragging at metaphase I (87.86% of 173 PMCs) and metaphase II (77.94% of 136 PMCs); chromosome lagging at anaphase I (59.32% of 59 PMCs) and anaphase II (50.00% of 48 PMCs); micronuclei at telophase I (61.60% of 125 PMCs) and telophase II (58.88% of 107 PMCs); and asynchrony of the chromosome during meiosis II (6.53% of 291 PMCs) (Figure 4b). The frequency of PMCs with chromosome behavior abnormalities in *mof1a-1* was higher than H1 at all observed stages (Table 2). These results indicate that *MOF1a* affects chromosome behavior during the meiosis process in neo-tetraploid rice.

### 2.5. MOF1a Defects Alter Expression Levels of Important Genes Involved in Tapetum and Meiosis in Neo-Tetraploid Rice

**Figure 5:**
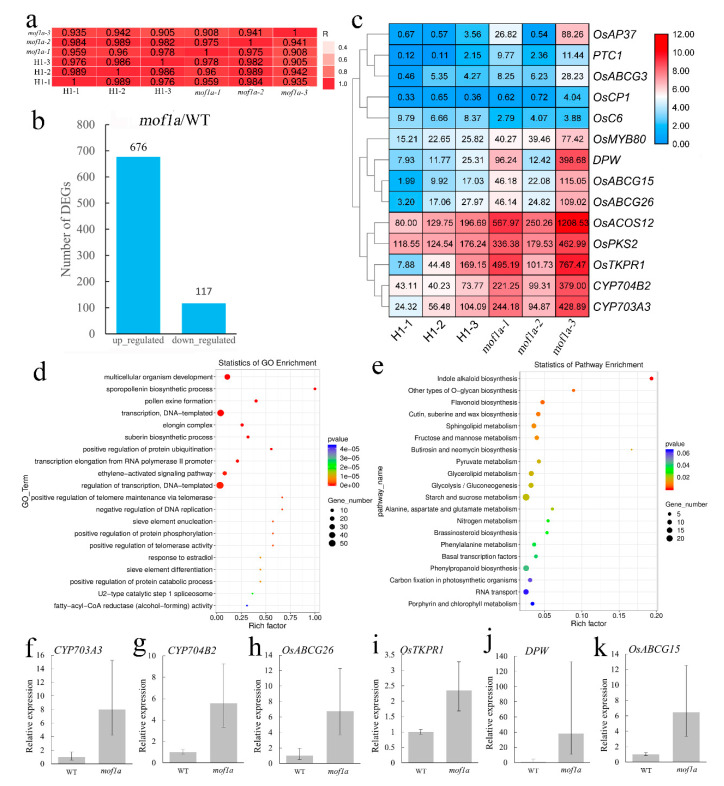
RNA-seq of *mof1a* and WT anthers during meiosis. (**a**) The Pearson correlation of RNA-seq between three biological replicates of *mof1a* and H1. (**b**) Number of DEGs that were identified from *mof1a*. (**c**) Hierarchical cluster analysis of average expression level (FKPM) of 14 tapetum related DEGs. Red indicates a relatively high expression level, while blue indicates a relatively low expression level. (**d**) Gene ontology enrichment of DEGs in *mof1a*. (**e**) KEGG pathway enrichment of DEGs in *mof1a*. (**f**–**k**) Expression levels of *CYP703A3* (**f**), *CYP704B2* (**g**), *OsABCG26* (**h**), *OsTKPR1* (**i**), *DPW* (**j**), and *OsABCG15* (**k**) in WT and *mof1a* anthers during microsporogenesis were analyzed using qRT-PCR. *Ubiquitin* gene was used as an internal control. The grey bars denote the mean expression of each gene (three independent replicates) and the error bars indicate the ±SD of three independent replicates.

To reveal genes regulated by *MOF1a*, RNA-seq of anthers during meiosis from a single plant of *mof1a* and WT was executed. A high degree of Pearson correlation (>0.905) was detected among three biological replicates of each material (Figure 5a). We identified 793 DEGs in *mof1a*, including 676 up- and 117 down-regulated genes (Figure 5b, Table S9). GO analyses revealed that these DEGs were enriched in key processes associated with anther development, such as sporopollenin biosynthetic process (GO: 0080110), pollen exine formation (GO: 0010584), and suberin biosynthetic process (GO: 0010345). KEGG pathway analysis demonstrated that these DEGs were involved in biosynthesis of indole alkaloid, O-glycan, flavonoid, cutin, suberin, and wax (Figure 5d,e). The GO and KEGG analyses showed that *MOF1a* was associated with important biosynthetic pathways during meiosis and pollen exine formation.

A total of 14 known genes associated with tapetum development were detected in DEGs, including *CYP703A3*, *CYP704B2*, *OsACOS12*, *DPW*, *OsABCG15*, *OsABCG26*, *OsABCG3*, *OsAP37*, *OsMYB80*, *PTC1*, *OsPKS2*, *OsTKPR1*, *OsCP1,* and *OsC6*. Among these genes, only *OsC6* was down-regulated in *mof1a,* while the remaining 13 DEGs were up-regulated. Six out of 13 up-regulated tapetal related-genes were selected for confirmation by qRT-PCR in WT and *mof1a*, which verified that all of *CYP703A3*, *CYP704B2*, *OsABCG26*, *OsTKPR1*, *DPW,* and *OsABCG15* were up-regulated in meiotic anthers of *mof1a* (Figure 5c,f,k).

In addition, these 793 DEGs in *mof1a* were further compared with 2987 meiosis-related genes (Table S10), which are specifically or preferentially expressed in PMCs, or were identified from defective meiosis process [10,26,27,32,33,34,35,40,41]. A total of 335 DEGs were meiosis-related genes (mDEGs), including 23 down- and 312 up-regulated mDEGs (Table S11). Among these 335 mDEGs, 29 mDEGs were differentially expressed in neo-tetraploid line H1 relative to low pollen fertility line LF, 50 mDEGs were differentially expressed in another neo-tetraploid line HF relative to LF, 146 mDEGs were specifically or preferentially expressed in PMCs [33,34,41], and 287 mDEGs were differentially expressed in meiotic anthers of autotetraploid lines with low fertility relative to corresponding diploid lines [4,10]. Moreover, nine mDEGs were up-regulated in low pollen fertility lines among three groups (HF/LF, H1/LF, and *mof1a*/H1), while all of them were differentially expressed in meiotic anthers between different ploidy lines (02428-4x/02428-2x or Taichung65-4x/Taichung65-2x) [4,10], including *LOC_Os03g61150*, *LOC_Os03g59880*, *LOC_Os08g28820* (S-phase kinase association protein 1, Skp1, family member), *LOC_Os01g04409*, *LOC_Os08g27220*, *LOC_Os02g50690*, *LOC_Os07g35370*, *LOC_Os01g13610*, and *LOC_Os11g47809* (*OsMT1a*, a type 1 metallothionein) (Table S12). These results indicated that a functional defect of *MOF1a* altered the expression levels of important meiotic genes in neo-tetraploid rice.

## 3. Discussion

### 3.1. MOF1a Plays a Key Role in the Pollen Development of Tetraploid Rice

Myeloblastosis (MYB) transcription factors are characterized by a highly conserved MYB DNA-binding domain. The MYB gene family contains at least 155 members in rice [42]. At least five MYB transcription factors play important roles during reproductive process in rice, including *OsGAmyb,* which modulates anther development in rice by regulating gibberellin [43], *OsMYB103* (*OsMYB80*), which regulates anther development by regulating the tapetum development and pollen wall formation [44,45], *OsMYB106,* of which the down-regulation causes pollen sterility and shortens plant height [46], *CSA,* which promotes rice pollen development and affects its sugar distribution via brassinosteroids [47,48], and *OsTDF1*, which is involved in tapetal development [49].

Recently, *MOF1* was found to encode a nucleoprotein with a MYB domain and two typical ethylene response factor-associated amphiphilic repression (EAR) motifs, and to regulate organ identity as well as spikelet determinacy in rice [36]. However, information about reproductive roles of *MOF1* are limited. In this study, we identified a new haplotype of *MOF1* from neo-tetraploid rice, in which some important SNPs were detected; as a result, it was named *MOF1a*. Importantly, *MOF1a* is a key gene that regulates pollen development of neo-tetraploid rice. The loss function of *MOF1a* caused abnormal tapetum degradation and abnormal meiosis process. Tapetal development and meiotic process have been well-investigated by many researchers [31,50]. Tapetal cells undergo degradation triggered by programmed cell death from meiosis to microspore stage, which is crucial to microspore development and pollen wall formation [51,52]. Thus, *MOF1a* played important role in pollen development of neo-tetraploid rice, especially affecting tapetum development and the meiosis process.

### 3.2. MOF1a May Affect the Gene Regulatory Network Associated with Tapetum Development or the Meiosis Process in Tetraploid Rice

In this study, loss function of *MOF1a* caused defective tapetum development with remarkable changes in the expression levels of 14 tapetal related-genes during meiotic anthers. Function of these 14 genes have been well understanding during anther development. *CYP703A3* and *CYP704B2* regulate 7-hydroxylated lauric acid generation and ω-hydroxylation of fatty acids [53,54]. *OsACOS12* involves in sporopollenin synthesis [55]. *DPW* participates in fatty alcohol synthesis for anther cuticle and sporopollenin biosynthesis [56]. *OsAP37* and *OsCP1* involves in programed cell death of tapetum [52,57]. Mutants of *OsPKS2*, *OsTKPR1*, *PTC1,* and *OsMYB80* lead to abnormal tapetum degeneration and most microspores lacked exines in mature anthers [45,57,58,59]. *OsABCG15*, *OsABCG26*, *OsABCG3,* and *OsC6* respond to transport lipidic molecules from tapetal cells [60,61,62]. These DEGs were involved in the biosynthesis pathway of important materials such as sporopollenin, the degeneration of tapetum, and the transportation of biosynthesis production from tapetum to microspore, which are essential for microspore subsequent development. The transcripts of these genes began to accumulate at early meiosis stages and accumulated a relative high level during meiosis, including *CYP703A3* (S8) [53], *CYB704B4* (S8) [54], *PTC1* (S8) [57], *OsABCG26* (S8) [62], and *WDA1* (S8) [63]. These results indicate that meiosis is an important stage for the initiation of tapetal biosynthesis, and suggest that *MOF1a* might regulate the expression levels of important tapetal related-genes to affect tapetum development. The expression pattern of tapetal genes are strictly controlled, and premature expression against their normal pattern may cause abnormal tapetum development. For example, a series of important tapetal gens abnormally up-regulated are expressed in *DTD*/*EAT1* mutant (*dtd*), including *WDA1*, *CYP703A3*, *OsAP37*, *OsGAmyb*, *OsCP1*, *PTC1*, *UDT1*, and *TDR*. As a result, *dtd* cannot normally start the programmed cell death of tapetum [64]. Similarly, 13 tapetal genes were up-regulated in meiotic anthers of *mof1a*. Interestingly, the expression levels of all differentially expressed tapetal genes gradually increased from S7 to S10 stage, including *CYP703A3*, *CYP704B2*, *OsGAmyb*, *OsC6*, *PTC1*, *OsCP1* [64], *OsABCG26* [62], *DPW* [56], *OsACOS12* [55], *OsABCG3* [60], *OsPKS2* [59], *OsAP37* [52], and *OsTKPR1* [58]. However, the expression level of *MOF1a* showed a peak in an early stage (S7) and gradually decreased from S7 to S10 stage in WT plants, which is opposite to the expression pattern of these tapetal genes. Recently, it was found that MOF1 protein has two EAR motifs with a strong repression effect on downstream genes [36]. Thus, *MOF1a* regulates the development of tapetum by repressing expression levels of tapetal biosynthesis related-genes in an early stage of anther development.

In addition, loss function of *MOF1a* also increased the frequency of PMCs with abnormal chromosome behaviors in seven stage of meiosis, and the most common abnormality was the straggle chromosome. In a previous study, many mutants of meiosis-related genes, such as *ZIP4*, *OsREC8*, *MRE11*, *OsSGO1*, *HEI10*, *OsDMC1*, and *OsMND1,* caused similar straggle chromosomes during meiosis process because of abnormal homologous synapsis or double strand break [65,66,67,68,69,70,71]. Disorder of microtube distribution is also considered a key reason of chromosome behaviors [9,72]. However, no known meiotic-related genes differentially expressed in *mof1a* anthers with defective meiosis, suggesting that *MOF1a* might regulate the meiosis process via another pathway, that most well-known meiotic genes might function upstream to regulate meiotic function of *MOF1a*, or that *MOF1a* mainly affect factors involved in the post-transcriptional regulation of these meiotic genes. As a first conjecture, scientists have performed abundant researches to identified meiosis-related genes, and we found 335 mDEGs in *mof1a*. In particular, there are nine mDEGs that were differentially expressed in more than three group samples with different meiotic process, suggesting that they may be associated with meiotic chromosome behaviors [4,10]. Among these nine genes, *LOC_Os08g28820* belongs to the Skp1 family, which is involved in Skp1-Cul1-F-box-protein (SCF) complex formation and regulates plant male meiosis process in Arabidopsis [73] and rice [74]; *LOC_Os01g04409* encodes an OsWAK receptor-like cytoplasmic kinase, which is a homolog of *OsWAK91*/*OsDEES1* regulating female gametophyte development [75]; *LOC_Os11g47809* (*OsMT1a*) encodes an metallothionein to regulate reactive oxygen species (ROS) levels, while another metallothionein OsMT2b controls ROS levels to effect pollen development [76]. All of these nine genes were up-regulated expressed in *mof1a*, suggesting that *MOF1a* might alter expression levels of a set of important meiotic related genes, particularly these nine genes, to regulate meiosis process in neo-tetraploid rice. Knowledge about these regulations is still limited, and more explorations are required to verify them in future studies.

## 4. Materials and Methods

### 4.1. Plant Material

One neo-tetraploid line with high fertility Huaduo1 (H1) and two recombinant inbred lines, i.e., HF with high fertility and LF with a low fertility, were used for RNA-seq. HF and LF were developed from the progenies of T428 (autotetraploid line with low fertility) × H1. T428 was developed from the chromosome doubling of diploid rice (*Oryza sativa* L. ssp. *japonica*), Linglun, by colchicine. H1 was also used as the receptor during CRISPR/Cas9 transgenes to generate *mof1a* mutants.

### 4.2. Evaluation of Seed Setting and Pollen Fertility

At least five plants of each material were harvested to measure their seed setting at maturity [1]. Pollen fertility was evaluated by 1% iodine-potassium iodide solution staining experiment [77]. The live pollen grains were subjected to 1% 2, 3, 5-Triphenyl-2H-tetrazolium chloride (TTC) solution at 31 °C for 1 h to evaluate pollen viability. In this case, the viable pollen grains would turn red while the devitalized one would keep primary color. Three spikelets from each plant (three plants for each material) were collected for pollen fertility or pollen viability experiments. Five pictures of each sample were taken under a microscope (Motic BA200) for the estimation of pollen fertility and viability.

### 4.3. RNA-seq Analysis

To determine that the anthers (pollen mother cells) are at meiosis stage, we measured the spikelet length. Our results showed that most of the pollen mother cells were at a meiosis stage when spikelet length was between 4.00 to 6.00 mm in H1, HF, and LF. Thus, the anthers were isolated from 4.00 mm to 6.00 mm spikelets. All the samples were kept in liquid nitrogen and stored at −80 °C for RNA isolation. Each sample was collected in three biological replications. The total RNA extraction, RNA-seq, and the evaluation of differentially expressed gene (DEG) were done as described by Guo et al. [1]. The DEGs were validated by qRT-PCR as described by Wu et al. [20]. The *Ubiquitin* gene was used as an internal control and all primers for qRT-PCR were designed by Primer Premier 5.0 and Primer-Blast software in NCBI (Table S13). The relative expression of qRT-PCR was calculated by the 2^−ΔΔ*C*T^ method [78].

### 4.4. Bioinformatics Analysis

Candidate genes were annotated according to GFF3 file of MSU7 genome [37]. The whole gene expression profile was predicted by using the Rice Expression Profile Database [39], Rice eFP expression profile analysis website [38], and GEO Profiles in NCBI. GO analysis of candidate genes was performed by using AgriGO tool [79]. Heat map diagram was drawn by TBtools [80].

Gene expression pattern of candidate genes was analyzed based on 84 RNA-seq datasets of tetraploid rice (included one control diploid line E252). These datasets were uploaded by our research group and publicly available for experiments under the access numbers SRP078960, PRJNA524942, PRJNA526133, and PRJNA576043 [1,18,24,25], including samples from anther (microsporogenesis, meiosis, before flowering), ovary (meiosis, before flowering, three day after flowering (DAF), five DAF), flag leaves (meiosis, before flowering, three DAF, five DAF), sheath (before flowering, three DAF, five DAF), spikelets (panicle length < 5 mm), and panicle branch (before flowering, five DAF). More detailed information is provided in Table S7.

### 4.5. Construction of CRISPR/Cas9 Vectors

We constructed the CRISPR/Cas9 vectors as described by Ma et al. [81]. For each candidate gene, two sgRNA expression cassettes (U6a and U6b promoters) were designed to target specific sites at the coding sequence to cause frameshift mutations (Figure 2a). The sgRNA sequence = target sequence + “GTTTTAGAGCTAGAAATAGCAAGTTAAAATAAGGCTAGTCCGTTATCAACTTGAAAAAGTGGCACCGAGTCGGTGC.” These two sgRNA expression cassettes were cloned into pYLCRISPR/Cas9Pubi-H by Golden Gate ligation. After transformation into *Escherichia coli* DH5-alpha, the recombinants were selected and sequenced. BioRun (Wuhan, China) was employed to complete the tissue culture of H1 and transform each vector into calli of H1 using *Agrobacterium*-mediated method. Transgenic seedlings were examined under natural field condition at the experimental station of South China Agriculture University, Guangzhou, China. In T_0_ generation, two primers (gRT14 and U6bT17) were used as molecular markers to identify T-DNA, which were specific to two sgRNA expression cassettes (Table S13). In T_1_ and T_2_ generation, two primers (Hyg-F and Hyg-R) were used as molecular markers for the hygromycin resistance gene to identify T-DNA (Table S13). Sanger sequencing was used for the phenotypic analysis of transgenic lines. T1MOF1 and t2MOF2 primers were used to detect the polymorphisms of target1 loci and target2 loci of *MOF1a* (Table S13).

### 4.6. Cytological Observation

Young panicles were collected from rice plants with 0.00 to 3.00 cm between their flag leaf cushion and the second-to-last leaf cushion, fixed in Carnoy solution (alcohol: acetic acid, 3:1 *v/v*) for more than 24 h, and then kept in 70% alcohol. Chromosome behavior and configuration were observed and analyzed as described by Wu et al. [4]. Meiosis stages were classified and explained according to He et al. [9].

A semi-thin sectioning was employed to characterize the anther wall development in HF and LF. The isolated anthers from spikelets in 70% alcohol were sequentially dehydrated in 85%, 90%, and 95% alcohol for 30 min. The dehydrated samples were added to penetrant (Basic resin: Activator = 50 mL: 0.5 g, Leica 7022 Histeresin Embedding Kit) with the same volume of 95% alcohol and stored at 4 °C for 10 h. The samples were transferred to pure penetrant and kept for 3 h. Then, 500 μL embedding medium (penetrant: Hardener= 15:1 *v/v*, Leica 7022 Histeresin Embedding Kit) and one sample were placed into a mold together. The solution with sample would condense after 30 min. Cross-sections (3 μm) were cut and stained with 1% Toluidine Blue O for several seconds and washed under the water. Then, the samples were observed under a microscope (Motic BA200).

A whole-mount eosin B-staining confocal laser scanning microscopy (WE-CLSM) was used to investigate the variations in mature embryo sac fertility. The spikelets of pre-flowering were collected and fixed in a Formaldehyde-Acetic acid-Alcohol solution (70% alcohol: acetic acid: formaldehyde = 18: 1: 1, *v/v*) for at least 24 h. Then, the samples were stored in 70% alcohol at 4 °C. The isolated ovaries were hydrated consecutively in 50%, 30%, and 10% alcohol and distilled water for 30 min. After an eosin B (10 mg/L in 4% sucrose solution) staining procedure for 10 h, the samples were dehydrated sequentially in 10%, 30%, 50%, 70%, 90%, and 100% (three times) alcohol for 30 min. The dehydrated samples were added to methyl salicylate with the same volume of alcohol and kept for 1 h. Finally, the samples were stored in pure methyl salicylate for 1 h and observed under WE-CLSM.

## Figures and Tables

**Table 1:** Seed setting and pollen fertility of Huaduo1 (H1), HF, and LF.

Materials	Seed Setting % ± SE	Pollen Fertility (PF) % ± SE
H1	77.94 ± 0.99A	95.15 ± 0.75A
HF	74.81 ± 2.02A	85.58 ± 0.86B
LF	8.58 ± 3.40B	54.82 ± 3.51C

H1, HF, and LF indicate a neo-tetraploid line, Huaduo1, a high pollen fertility recombinant inbred line, and a low pollen fertility recombinant inbred line, respectively. Least significant difference (LSD) was used in the multiple comparison for each trait. Different letters between two samples indicated significant differences (*p* value < 0.01).

**Table 2:** Frequency of pollen mother cells with abnormal anther chambers or abnormal chromosome behavior during meiosis in WT (H1) and *mof1a.*

Item	Stage	WT		*mof1a*	
		Number	Abnormal (%)	Number	Abnormal (%)
Anther sections	S8a	178	2.81	39	74.36
	S8b	147	0.00	103	72.82
	S10	98	0.00	44	100.00
Meiosis process	Diakinesis	145	16.55	104	47.12
	Metaphase I	234	12.39	173	87.86
	Anaphase I	57	21.05	59	59.32
	Telophase I	222	7.21	125	61.60
	Metaphase Ⅱ	257	19.84	136	77.94
	Anaphase Ⅱ	53	3.77	48	50.00
	Telophase Ⅱ	215	4.65	107	58.88
	Asynchrony in meiosis Ⅱ	525	6.29	291	6.53

S8a, S8b, and S10 indicate anther development stages. S8a and S8b represents the meiosis process. S10 represents the single microspore stage.

## Supplementary Materials

The following are available online at https://www.mdpi.com/1422-0067/21/20/7489/s1, Figure S1: The Pearson correlation between three biological replicates of RNA-seq in HF, LF, and H1. Figure S2: Predicted expression pattern of *MOF1*. Figure S3: Expression pattern analysis of *MOF1* by RNA-seq data. Figure S4. Transcription level of *MOF1a* in WT and *mof1a* plants by qRT-PCR. Table S1: Average RNA-seq data quality among three biological replicates in H1, HF, and LF. Table S2: Differentially expressed genes (DEGs) between H1 and LF. Table S3. Differentially expressed genes (DEGs) between HF and LF. Table S4. Common differentially expressed genes (coDEGs) between HF and H1. Table S5: Eight candidate genes related to the anther development. Table S6: The SNPs and InDels of *MOF1a* in H1. Table S7: Detailed information about the expression pattern analysis of *MOF1* by RNA-seq data (Figure S5). Table S8: The seed setting and mutant information of WT and *mof1a*. Table S9: Differentially expressed genes (DEGs) between *mof1a* and WT. Table S10: Meiosis-related gene set. Table S11: Differentially expressed meiosis-related genes in *mof1a*. Table S12: Differentially expressed meiosis-related genes (mDEGs) overlapped in *mof1a*/H1 and LF/H1-HF. Table S13: Primers used in this study.
