# Supplementary material for: Transcriptome and Gene Editing Analyses Reveal MOF1a Defect Alters the Expression of Genes Associated with Tapetum Development and Chromosome Behavior at Meiosis Stage Resulting in Low Pollen Fertility of Tetraploid Rice
Source: Int J Mol Sci. 2020 Oct 11;21(20):7489. doi: 10.3390/ijms21207489 (PMC7589589; doi:10.3390/ijms21207489)
Supplement: Supplementary file 1 [file ijms-21-07489-s001.zip › Lu-P1-Supplementary+file+1+2020-08-14(1).pdf]

Transcriptome and gene editing analyses reveal MOF1a defect alters the expression of genes associated with tapetum development and chromosome behavior at meiosis stage resulting in low pollen fertility of tetraploid rice

Zijun Lu, Xiaotong Guo, Zhiyu Huang, Juan Xia, Xiang Li, Jinwen Wu, Hang Yu, Muhammad Qasim Shahid, Xiangdong Liu

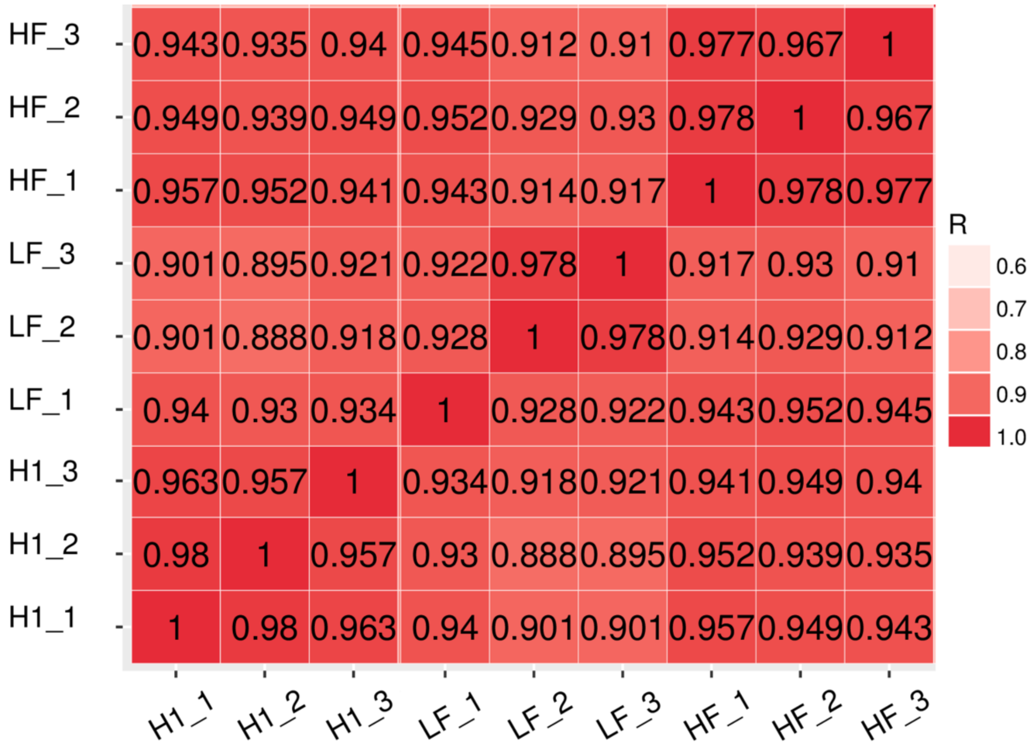

Figure S1 The Pearson correlation between three biological replicates of RNA-seq in HF, LF and H1. LF, HF and H1 indicate low pollen fertility recombinant inbred line, high fertility recombinant inbred line and neo-tetraploid rice, respectively

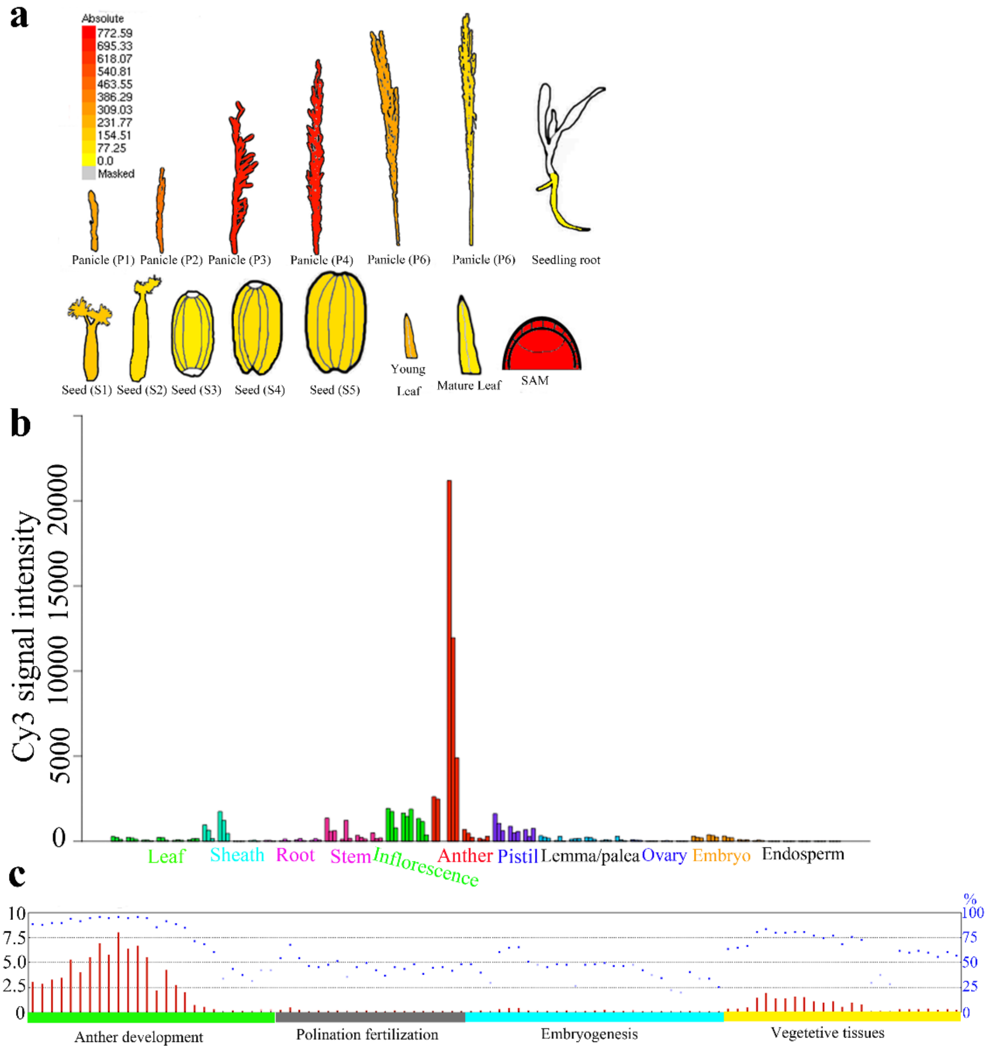

Figure S2 Predicted expression pattern of *MOF1*. (**a**) Expression pattern of *MOF1* in Rice eFP Browser. SAM, shoot apical meristem. Red indicates relatively high expression level. *MOF1* highly expressed during panicle development. (**b**) Expression pattern of *MOF1* in RiceXPro. *MOF1* highly expressed in anther. (**c**) Expression pattern of *MOF1* in GEO Profiles of NCBI. *MOF1* highly expressed during anther development.

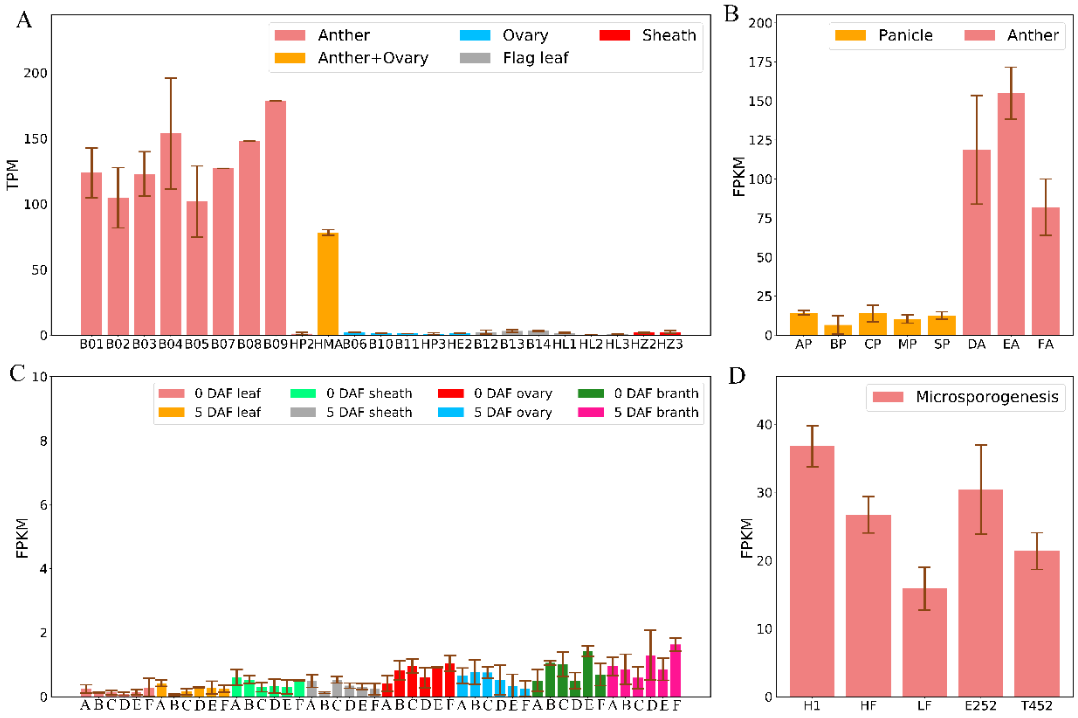

Figure S3 Expression pattern analysis of *MOF1* by RNA-seq data. (**a**) Expression level of *MOF1* in anther, ovary, anther + ovary, flag leaf, and sheath. (**b**) Expression level of *MOF1* in panicle (length < 5 mm) and anther. (**c**) Expression level of *MOF1* in tetraploid rice, DAF represents days after-flowering. (**d**) Expression level of *MOF1* in anthers (microsporogenesis). The indexes of X-axis indicate different lines of tetraploid rice (E252 is diploid rice) and detailed information is provided in Supplementary file 2: Table S7. The error bars indicate the ±SD of three independent replicates

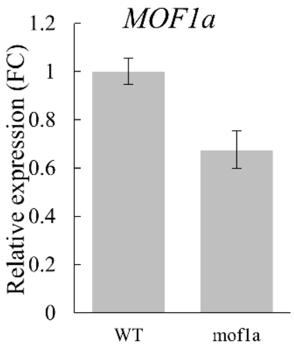

Figure S4 Transcription level of *MOF1a* in WT and *mof1a* plants by qRT-PCR. FC, fold change. *Ubiquitin* gene was used as an internal control. The grey bars denote the mean expression of three independent replicates and the error bars indicate the ±SD of three independent replicates
